# Single chain Fab (scFab) fragment

**DOI:** 10.1186/1472-6750-7-14

**Published:** 2007-03-08

**Authors:** Michael Hust, Thomas Jostock, Christian Menzel, Bernd Voedisch, Anja Mohr, Mariam Brenneis, Martina I Kirsch, Doris Meier, Stefan Dübel

**Affiliations:** 1 Abteilung Biotechnologie, Institut für Biochemie und Biotechnologie, Technische Universität Braunschweig, Spielmannstr.7, 38106 Braunschweig, Germany; 2 Novartis International AG, CH-4002 Basel, Switzerland; 3 Boehringer Ingelheim Pharma GmbH & Co. KG, Birkendorfer Straße 65, 88397 Biberach/Riß, Germany; 4 Biochemisches Institut, Universität Zürich, Winterthurerstrasse 190, 8057 Zürich, Switzerland; 5 Institut für Molekulare Biowissenschaften, Johann Wolfgang Goethe Universität Frankfurt, Max-von-Laue-Str. 9, 60438 Frankfurt am Main, Germany

## Abstract

**Background:**

The connection of the variable part of the heavy chain (VH) and and the variable part of the light chain (VL) by a peptide linker to form a consecutive polypeptide chain (single chain antibody, scFv) was a breakthrough for the functional production of antibody fragments in *Escherichia coli*. Being double the size of fragment variable (Fv) fragments and requiring assembly of two independent polypeptide chains, functional Fab fragments are usually produced with significantly lower yields in *E. coli*. An antibody design combining stability and assay compatibility of the fragment antigen binding (Fab) with high level bacterial expression of single chain Fv fragments would be desirable. The desired antibody fragment should be both suitable for expression as soluble antibody in *E. coli *and antibody phage display.

**Results:**

Here, we demonstrate that the introduction of a polypeptide linker between the fragment difficult (Fd) and the light chain (LC), resulting in the formation of a single chain Fab fragment (scFab), can lead to improved production of functional molecules. We tested the impact of various linker designs and modifications of the constant regions on both phage display efficiency and the yield of soluble antibody fragments. A scFab variant without cysteins (scFabΔC) connecting the constant part 1 of the heavy chain (CH1) and the constant part of the light chain (CL) were best suited for phage display and production of soluble antibody fragments. Beside the expression system *E. coli*, the new antibody format was also expressed in *Pichia pastoris*. Monovalent and divalent fragments (DiFabodies) as well as multimers were characterised.

**Conclusion:**

A new antibody design offers the generation of bivalent Fab derivates for antibody phage display and production of soluble antibody fragments. This antibody format is of particular value for high throughput proteome binder generation projects, due to the avidity effect and the possible use of common standard sera for detection.

## Background

The production of functional antibody fragments in *E. coli *was first described by Skerra and Plückthun [1]. Key to this success was production in the periplasm, where the oxidizing environment allows the formation of disulphide bonds. Later, the linkage of the variable regions by a 15–25 amino acid linker of both Fv chains improved the expression of antibody fragments in *E. coli *[2,3]. However, these so called single chain fragment variable (scFv) have the tendency to form aggregates and are relatively unstable over longer periods of time [4]. Furthermore, some scFvs show a reduced affinity of up to one order of magnitude compared to the corresponding Fab fragments [5]. Only in rare cases have scFvs with a higher affinity than the associated Fab been found [6]. Because they are double the molecular size, and require the production and connection of two different polypeptides with a disulphide bond, folding and assembly of Fab fragments in the periplasm of *E. coli *is less efficient than for scFvs [7]. A further disadvantage of Fab fragments is the tendency of light chains to form homo-dimers, which are known as Bence Jones proteins [8,9]. Advantages of Fab fragments are their high stability in long term storage [10] and their compatibility with common detection antisera without the need for a re-engineering step [11]. An antibody design combining stability and assay compatibility of Fab fragments with high level bacterial expression of single chain Fv fragments would be desirable. The desired antibody fragment should be both suitable for expression as soluble antibody in *E. coli *and antibody phage display.

Currently, most recombinant antibody fragments are generated by antibody phage display. Phage display technology is based on the groundbreaking work of Smith [12]. Antibody phage display was first described by Huse *et al*. [13] for the phage Lambda and by McCafferty *et al*. [14] for the M13 phage. However, practical use was only achieved by uncoupling antibody gene replication and expression from the phage life cycle by locating them on a separate plasmid (phagemid) to improve genetic stability, handling, and screening of antibody libraries [15-18]. So far, naive scFv antibody libraries with a theoretical diversity of up to 10^11 ^independent clones [19] and Fab antibody libraries with a size of 3.5 × 10^10 ^clones [20] have been generated as molecular repertoires for phage display selections (overview given by Hust and Dübel [21]). Antibody phage display is a key technology for the generation of human recombinant antibody fragments for therapy and diagnostics [22].

Here, we demonstrate, that the introduction of a polypeptide linker between Fd fragment and light chain, resulting in the formation of a single chain Fab fragment (scFab), can lead to improved production of antibody fragments. We tested the impact of various linker lengths and the presence of the disulphide bond on both the display efficiency on phage and the yield of soluble antibody fragment production in both prokaryotic and eukaryotic cells.

## Results

### Construction of the pHAL vectors with different antibody formats

**Figure 1:**
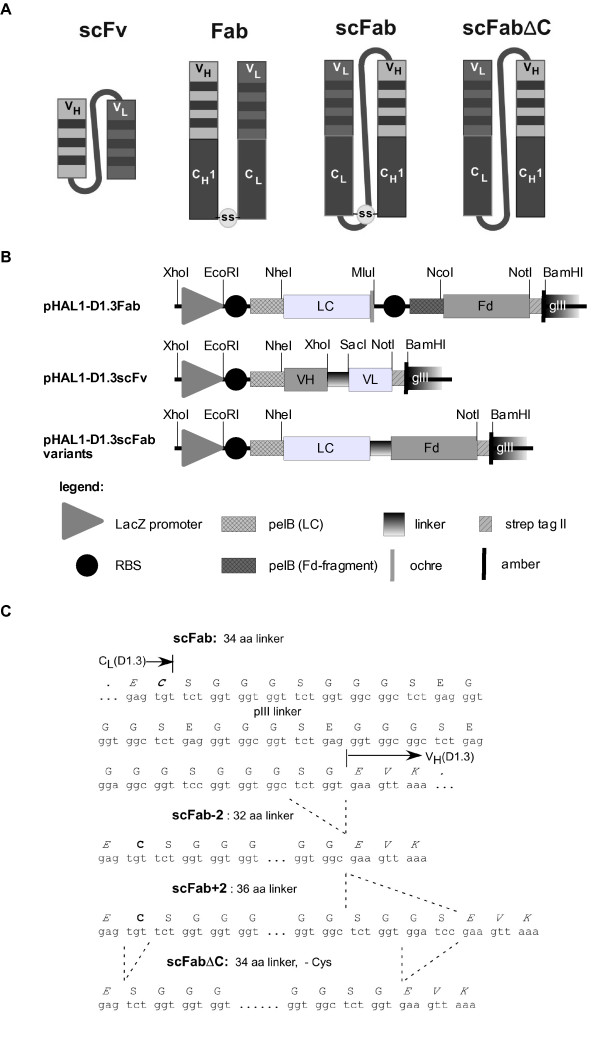
Vectors constructed for this study. **A **illustration of the different antibody formats, **B **the pHAL1-D1.3 vectors, **C **linker sequences of the scFab variants. Abbreviations: lacZ promoter: promoter of the bacterial lac operon; RBS: ribosome binding site; pelB: signal peptide sequence of bacterial pectate lyase, mediating secretion into the periplasmic space; VH: variable fragment of the heavy chain; LC: light chain; ochre: ochre stop codon; amber: amber stop codon; *strep*-tag II: synthetic tag binding to streptactin. The elements of the inserts are not drawn to scale.

The mouse anti hen egg white lysozyme antibody D1.3 [23,24] was reformated into a scFab with a 34 amino acid linker, a scFab-2 with a 32 amino acid linker, a scFab+2 with a 36 amino acid linker and a scFab variant with a 34 amino acid linker where the cysteins connecting both antibody chains were deleted (scFabΔC). Linker length was chosen to cover the space between the LC carboxyterminus and the aminoterminal end of the Fd fragment, as determined from X ray crystallographic data of Fab fragments. To reduce the risk of adverse effects of the linker sequence on the yield or folding, an intra-domain linker sequence from the phage protein pIII was duplicated and inserted. The antibody fragments were cloned into the vector pHAL1 [9]. Vectors and linkers are given in figure 1.

### Production of antibody phage

**Figure 2:**
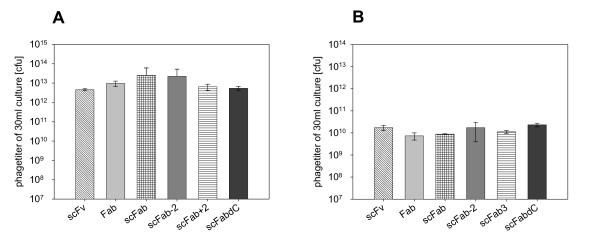
Comparison of antibody phage titres (cfu) obtained from 30 mL of culture of six different D1.3 antibody fragment variants. Packaged **A **with M13K07, **B **with Hyperphage. Mean value and standard deviations are from three completely independent experiments.

For evaluation of the antibody formats in phage display, different pHAL1-D1.3 phagemids were packaged using either Hyperphage or M13K07. As decribed, when using the helperphage M13K07 (Fig. 2a) observed phage yields were higher than when using Hyperphage (Fig. 2b). When using M13K07, the yield was between 5 × 10^12 ^and 1 × 10^13 ^phage per 30 mL culture, whereas 5 × 10^9 ^to 5 × 10^10 ^phage per 30 mL culture were produced with Hyperphage. The phage productions showed only marginal differences between the antibody designs when using M13K07 or Hyperphage.

### SDS-PAGE analysis of antibody phage

**Figure 3:**
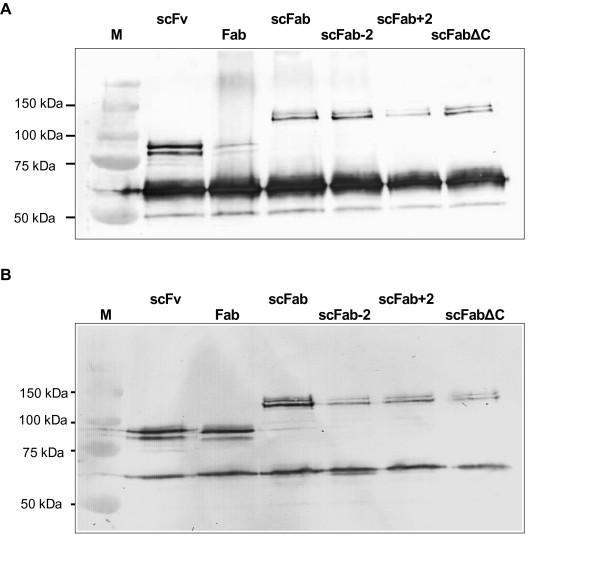
Immunoblot of antibody phage presenting different formats, produced with either M13K07 or Hyperphage. **A **1 × 10^11 ^D1.3 antibody phage produced with M13K07 were separated, **B **3 × 10^8 ^D1.3 antibody phage produced with Hyperphage were separated. Phage were separated on a reducing 10 % SDS-PAGE, pIII was detected using mouse mAb anti-pIII.

D1.3 antibody phage preparations generated with M13K07 or Hyperphage were separated by SDS-PAGE under reducing conditions and blotted onto PVDF membranes. pIII was visualised by immunostaining using a monoclonal mouse anti-pIII antibody. pIII has a calculated molecular mass of 42,5 kDa, but it runs at an apparent molecular mass of 65 kDa in SDS-PAGE [15,25]. As expected, with phage produced using M13K07, the pIII band at 65 kDa dominated, especially for Fab, reflecting a low amount of fusion protein obtained in monovalent display (Fig. 3a). The antibody-pIII fusion was more prominent for the phage generated with Hyperphage and is present in amounts almost equal to the unfused pIII (Fig. 3b). The display efficiency of all scFabs variants was better than that of the Fab fragments when using M13K07.

### Antigen ELISA of antibody phage

Antigen binding of phage presenting the different antibody formats was determined by ELISA on lysozyme. 5 × 10^8 ^phage/well were applied when M13K07 was used for phage rescue, whereas 10^7 ^phage/well were used after packaging with Hyperphage to compensate the known differences in antibody presentation efficiency of both systems [9,26,27].

**Figure 4:**
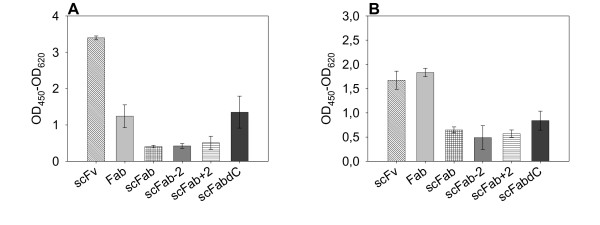
Antigen binding phage ELISA of phage presenting D1.3 antibody fragment variants. **A **5 × 10^8 ^phage produced with M13K07, **B **10^7 ^phage produced with Hyperphage. Mean values and standard deviations of three completely independent experiments are given. The absorbance of scattered light at 620 nm was subtracted from the absorbance at 450 nm. The background signal of the antigen incubated with the detection antibody mAb anti-M13 HRP was subtracted. Antigen: 100 ng/well lysozyme. The D1.3 antibody phage were detected using mAb anti-M13 conjugated with HRP.

In the case of phage packaged using M13K07 (Fig. 4a) the scFv format showed best antigen binding. The Fab and scFabΔC fragments bound the antigen approximately half as good as the scFv. The presentation of functional antibody fragments on the phage surface was weaker for the other scFab variants. The results for Hyperphage packaged phage (Fig. 4b) were different. Here, the Fab and scFv fragments were presented best as functional proteins on phage, whereas the scFab variants were one third as good. However, the scFabΔC was the best variant of the scFab variants.

### Production of soluble antibody variants using the phage display vector pHAL1

**Figure 5:**
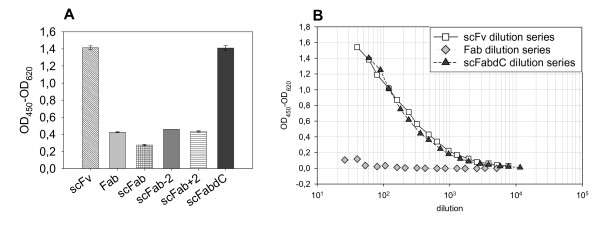
Antigen binding ELISA of soluble antibody fragments produced in *E. coli *using pHAL1 with 1 μg/well lysozyme coated per well. **A **20 μL periplasmic fractions from production in MTPs of scFv, Fab and four scFab constructs were applied, the D1.3 antibodies were detected using mouse mAb anti-Strep-Tag (1:10000), **B **serial dilutions of Protein L purified equimolar amounts of scFv, Fab and scFabΔC were applied, D1.3 antibody fragments were detected using Protein L conjugated to HRP (1:10000)

In order to investigate the production of soluble D1.3 scFab antibody fragments using phage display vectors, 20 μL aliquots of supernatant from an overnight expression of *E. coli *strain XL1-Blue MRF', transformed with pHAL1-D1.3 constructs in MTPs, were analyzed by antigen ELISAs using mAb anti-strep tag for detection (Fig. 5a). This assay rates the production and binding of the antibody fragments in combination. The highest signals corresponding to the best yield of functional antibody fragments were obtained with the scFv and the scFabΔC variants, indicating that these formats were produced with a high fraction of correctly folded antibody fragments. The deletion of the cysteins in the scFab led to an increased yield of functional antibody fragments. Additionally, the three antibody fragments were produced in 100 mL scale in *E. coli *and purified using Protein L. Serial dilutions of equimolar fractions, estimated by SDS-PAGE, of scFv, Fab and scFabΔC were used to compare the content of functional protein by lysozyme antigen ELISA (Fig. 5b). While same amounts of scFv and scFabΔC bound the antigen almost equal, the Fab fragments showed about 100× less antigen binding.

### Production of soluble antibody variants using the *E. coli *expression vector pOPE101-XP

**Figure 6:**
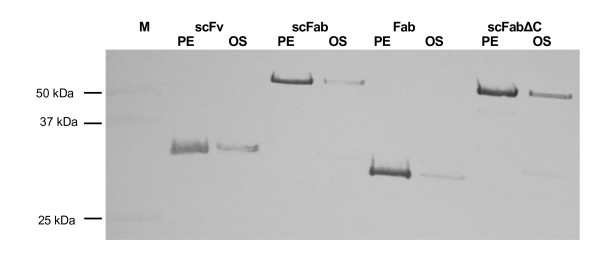
Immunoblot of soluble antibody fragments produced using the *E. coli *expression vector pOPE101. 20 μL aliquots of E. coli periplasmic fractions were separated on reducing 10% SDS-PAGE, pIII was detected using mouse mAb anti-myc tag and goat anti-mouse IgG AP. Abbreviations: PE: periplasmic fraction; OS: osmotic shock preparation.

Production of different D1.3 antibody fragments was also tested using the dedicated *E. coli *expression vector pOPE101-XP. 20 μL aliquots of the periplasmic fraction and the osmotic shock fraction were analysed by separation on a reducing 10% SDS-PAGE and immunoblot. The antibody fragments were detected using mAb mouse anti-myc tag antibody. The estimated relative molecular mass of all antibody fragments corresponded with the relative molecular mass of the scFab and scFabΔC including all tags of about 50.8 kDa, respectively 50.6 kDa, the scFv with a relative molecular mass of about 26.7 kDa and the Fd fragment of the Fab with a relative molecular mass of about 24,7 kDa (Fig. 6).

Afterwards, the antibody fragments were purified by IMAC followed by protein L affinity chromatography. Purified samples of scFv, Fab, scFab and scFabΔC were analyzed by surface plasmon resonance (SPR) to determine the affinity of the different antibody variants. Following affinities were measured: scFv: 1,04 × 10^-8 ^M; Fab 1,91 × 10^-8 ^M; scFab: 3,46 × 10^-8 ^M; scFabΔC: 4,45 × 10^-8 ^M. All measured affinities were in a similiar range, indicating that the newly introduced linker does not inhibit or impair binding to the antigen.

**Figure 7:**
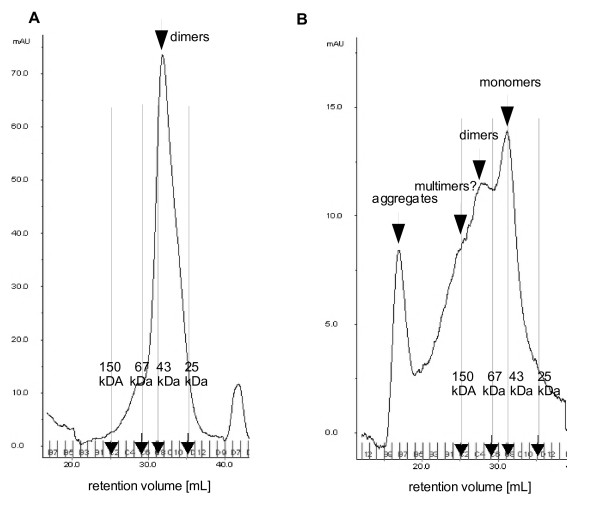
Size exclusion chromatography analysis of soluble antibody fragments produced using the *E. coli *expression vector pOPE101. 50 μg purified antibody fragments of the **A **scFv, or **B **scFabΔC format were separated on superdex 200. Calibration was done with Chymotrypsinogen (25 kDa), Ovalbumin (43 kDa), Albumin (67 kDa) and IgG (150 kDa).

**Figure 8:**
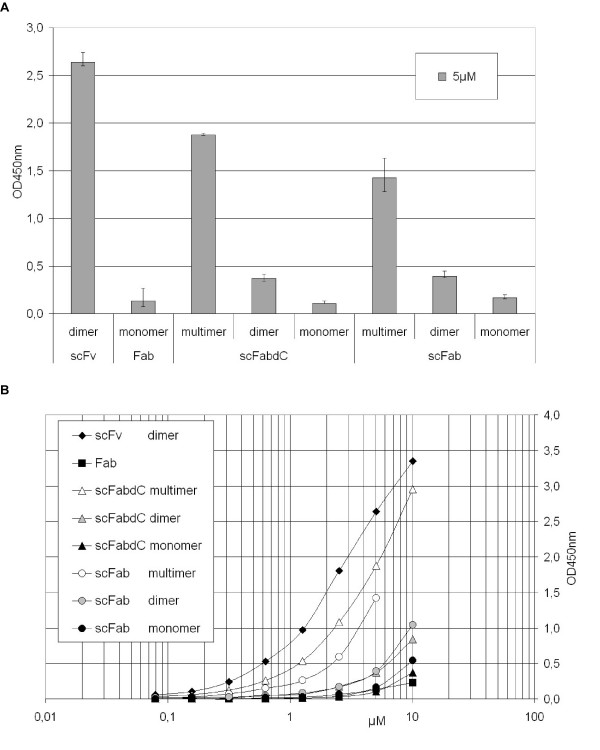
Antigen binding ELISA of soluble antibody fragments produced in *E. coli *using pOPE101 with 1 μg/well lysozyme coated per well. **A **5 μM gelfiltration purified fractions of scFv, Fab and four scFab constructs were applied per well, D1.3 antibody fragments were detected using mAb anti-myc tag (1:25), the ELISA measurements were done in triplicate. **B **ELISA signals of serial dilutions of the different fractions of the antibody fragments.

Monomers and multimers of the antibody fragments were separated by size exclusion chromatography. The gel filtration results are shown exemplarily for the scFv (Fig. 7a) and scFabΔC (Fig. 7b). The major peak corresponds to the size calculated for the scFabΔC monomers, almost the same amount represents dimers. The Fab existed as monomer and the other scFabs also showed monomer, dimer and multimer fractions (data not shown). The separated monomers, dimers and multimer fraction of all different antibody fragments were analysed by antigen binding ELISA on lysozyme with 5 μM antibody fragments (Fig. 8a) and with a serial dilution of the antibody fragments (Fig. 8b). The scFv was present as a dimer, and this antibody fragment showed the best binding to lysozyme. Multimers of scFab and scFabΔC also showed good antigen binding and the monomeric Fab, scFab and scFabΔC fractions bound weaker to lysozyme. As expected, the multimers bound better than the dimers and the dimers bound better than the monomers to the antigen.

### Production of scFab in *P. pastoris*

**Figure 9:**
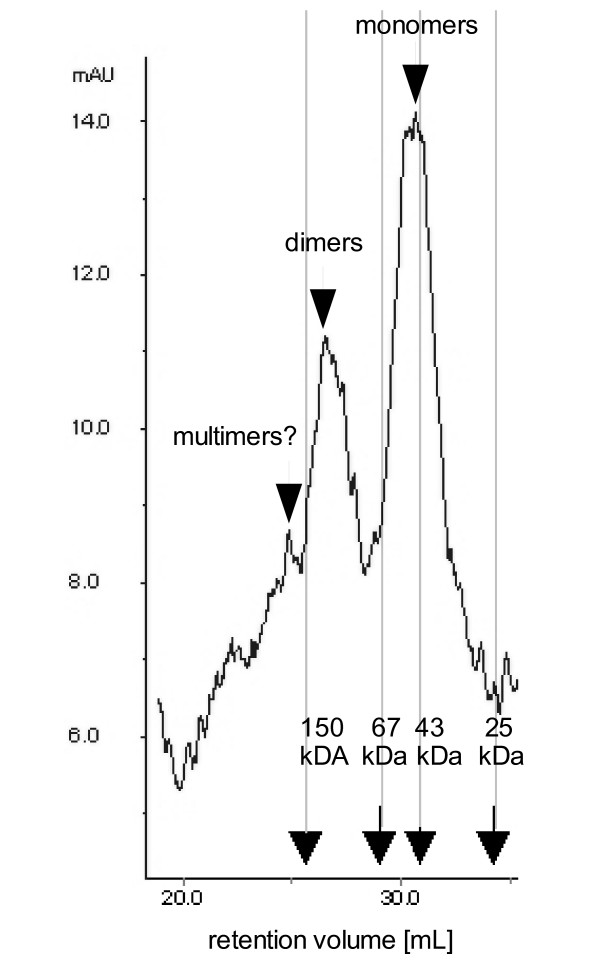
Size exclusion analysis of soluble antibody fragments produced in *P. pastoris*. 500 μg purified antibody fragments of the scFabΔC format were separated on Superdex 200.

**Figure 10:**
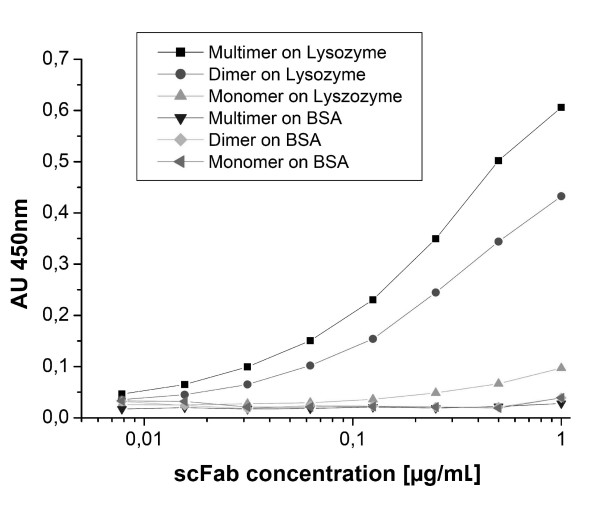
Antigen binding ELISA of soluble antibody fragments produced in *P. pastoris *with 100 ng/well lysozyme or BSA coated per well. A serial dilution of gel filtration purified fractions of scFabΔC ranging from 8 ng/mL to 1 μg/mL were applied, the D1.3 antibody fragments were detected using Protein L HRP conjugate (1:10000). Measurements were done in duplicate.

The D1.3scFabΔC was expressed in *P. pastoris *to investigate compatability of the scFab format with an eukaryotic expression system. Antibody fragments were purified by protein L affinity chromatography and analysed by size exclusion chromatography (Fig. 9). The scFabΔC showed a strong tendency to form dimers. The gel filtration fraction of monomeric, dimeric and presumably multimeric scFabΔC were analysed by ELISA on lysozyme (Fig. 10). As expected, due to the avidity effect, the multimers showed strongest binding followed by dimers and monomers.

## Discussion

So far, two antibody formats, Fabs and scFvs, have dominated antibody phage display and the production of soluble antibody fragments in *E. coli*. In this study, to test whether it is possible to combine the advantages of both formats, various single chain Fab (scFab) constructs were generated and evaluated. In scFab, the light chain and the Fd fragment of a Fab fragment are connected by a polypeptide linker derived from the pIII of filamentous phage M13. Three scFab variants with different linker length from 32 to 36 amino acids and one 34 amino acid variant with a deleted intermolecular disulphide bond (scFabΔC) were compared to scFv and Fab using the lysozyme binding antibody D1.3 [23,24] as a model antibody. In mammalian cells, an antibody construct using a 30 aa Gly-Ser linker to combine the HC fragment and the LC has succesfully been produced to generate a single chain IgG [28]. The scFab designs presented in this study, in contrast, were evaluated both for their compatibility with antibody phage display and production of soluble antibody fragments in microbial production systems. For the latter, in the prokaryotic expression system *E. coli*, both the phage display phagemid pHAL1 [9] and the *E. coli *expression vector pOPE101 [29] were tested. Additionally, the production in the eukaryotic expression system using the yeast *P. pastoris *was demonstrated.

The applicability of the new antibody designs for antibody phage display is of major importance, as antibody phage display is currently a major technology for the generation of human antibodies for research, diagnostic and therapy (for reviews see [11,22,30]. Display of the different antibody formats on phage was evaluated using the two most frequently used helperphage, the standard M13K07 and the pIII deficient Hyperphage [26,27,31], which enforces polyvalent display on filamentous phage. As described before, when the helperphage M13K07 was used, phage yields were higher compared to Hyperphage. This results from the lack of pIII when Hyperphage is used, leaving the less well produced pIII fusion with the antibody fragment as the sole source of this coat protein. The fusion of pIII to different scFab variants did not significantly inhibit phage assembly with both helperphage, indicating that all of the tested antibody designs are compatible with phage assembly. The length of the polypeptide linker used in the scFab variants had no influence on phage packaging and antigen binding. In contrast, significant benefit was achieved by removing the cysteins from the carboxyterminus of Fd and LC, adding to the body of evidence on the adverse effect of cysteins on the production of proteins in *E. coli *[29].

The phage display vector pHAL1 can also be used for production of soluble antibody fragments. This facilitates the analysis of individual clones after the panning procedure, because no recloning steps are necessary. Fractions of soluble antibodies are analysed by ELISA on enzyme rating the production and binding of the antibody fragments in combination. Here, the scFv and scFabΔC designs showed superior antigen binding compared to Fab and scFab. Consistent to the results obtained from the antigen ELISA using antibody phage, the length of the polypeptide linker used in the scFab variants had no influence on the antigen binding. Again, the cysteins connecting CH1 and CL chains in Fab and scFabs had also a negative effect on the production of functional antibody fragments. For further analysis, the scFv, Fab, scFab with 34 amino acid linker and the scFabΔC were produced in *E. coli *using the expression vector pOPE101 [29] and analysed in detail. The measured affinities by surface plasmon resonance (SPR) showed similiar results for all antibody variants and are in contrast to the results of the functional ELISAs. The scFab variants were found as monomers, dimers and multimers, the scFv was only found as dimer, and the Fab occured only as monomer. The multimeric and dimeric antibody fragment fractions showed better antigen binding compared to the monomeric states, indicating the expected avidity benefit. The results obtained for *E. coli *could be reproduced by analysis of scFabΔC produced in *Pichia pastoris*. This yeast expression system is of growing importance for antibody production because a glycosylation of full size antibodies identical to that in humans was achieved with glycoengineered *P. pastoris *lines [32].

**Figure 11:**
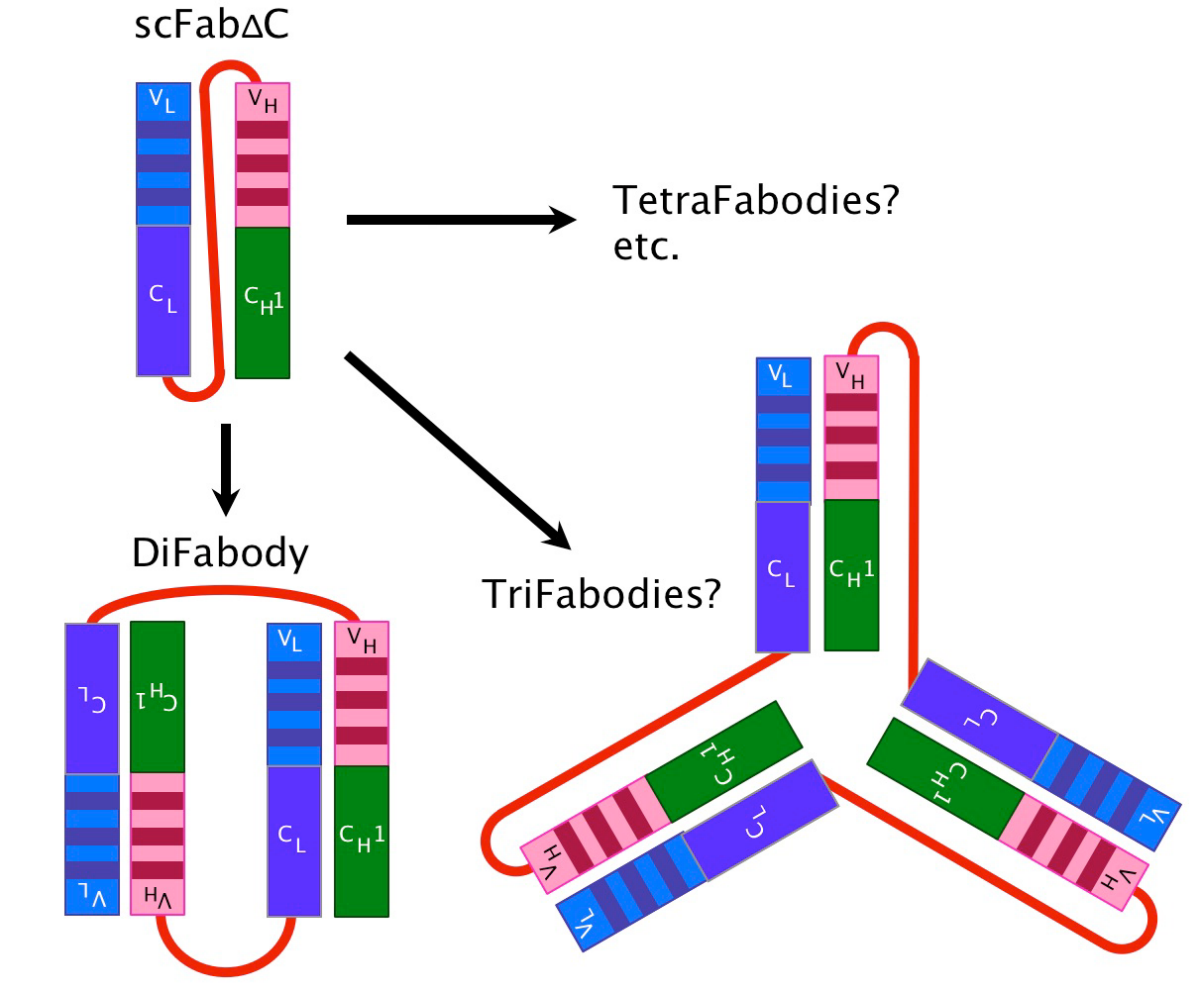
Illustration of scFabΔC and the multimerisation forms DiFabody and TriFabody.

In summary, the results show that scFab variants which are fully compatible with antibody phage display and superior to Fab fragments can be designed. Second, the scFabs, in particular the delta cysteine variant (scFabΔC), compensated for some of the disadvantages of soluble Fab production in *E. coli*. However, in phage display, we see the major advantage of the scFab format in the context of an optimised procedure for the generation of large amounts of binders on a proteome wide scale [30]. Here, a streamlined process has to be developed and optimised for significantly different parameters when compared to the current "industry standard" methods applied to the selection of therapeutic antibody candidates [11]. The improved display and stability of a robust Fab-size fragment that is compatible to widely used detection antisera may be a significant advantage. Further, the dimerisation offers an apparent affinity increase by avidity in most assays, without it being necessary to re-engineer or subclone the antibody's binding site. Otherwise the occurance of different states in the scFab variants makes it difficult the calculate the affinity. Furthermore, a mixture of states can not be used for clinical purposes. The association of two scFv polypeptide chains to form a noncovalent dimer (diabody) has been described frequently [33-38]. We observed a similar capability of the scFab polypeptides, resulting in a protein fraction with double the molecular mass and increased binding due to the avidity provided by two binding sites. This molecular species was shown to be formed independently from the production host and its chaperone equipment, in both the gram-negative bacterium *E. coli *and the yeast *Pichia pastoris*. Further, even TriFabodies, TetraFabodies and larger assemblies of the same polypeptide chain seem to be formed in analogy to the Triabodies and Tetrabodies described for the scFv constructs (Fig. 11) [38]. TetraFabodies may be favoured among these multimers for topological reasons. The size exclusion chromotography data of both the *E. coli *and *P. pastoris *derived materials strongly encourage the further research and characterization of these molecular species. It should be noted that in analogy to the scFv polypeptides, the formation of bispecific DiFabodies containing two different sets of V regions seems easily achievable using designs homologous to those described for single chain Fvs.

## Conclusion

The novel antibody design scFabΔC can be used for the production of soluble antibody fragments in *E. coli *and *Pichia pastoris *and is compatible with the phage display technology. In particular, it allows the use of common standard detection secondary sera, thus avoiding additional anti-tag antibody. Further, reagents with high apparent affinity can be created due to the avidity effects of DiFabodies without any subcloning steps. This advantage is of particular value for high throughput proteome binder generation projects.

## Methods

### pHAL scFab phage display vectors

Standard cloning procedures were performed according to Sambrook and Russell [39]. The pHAL vectors containing the different antibody formats were derived from the phagemid vector pHAL1 [9]. This vector allows both antibody phage display and expression of soluble antibody fragments due to an amber stop codon between the antibody coding region and *gIII*.

Construction of pHAL1-D1.3scFv (Fig. 1) was done by assembly PCR. VL fragment and VH fragment DNA were amplified from pHAL1-D1.3Fab [9] and assembled by a third PCR. The PCR product was cloned into the *Nhe*I and *Not*I site of pHAL1. For the construction of pHAL1-D1.3scFab, the coding region of the 34 amino acids glycine-serine linker (Fig. 1b) was amplified from the M13 geneIII (*gIII*) of pHAL1-D1.3Fab, by assembly PCR. The PCR product was cloned into the *Bss*HII and *Pst*I site of pHAL1-D1.3Fab. Two codons were deleted by PCR primer directed mutagenesis for the construction of pHAL1-D1.3scFab-2 and the PCR product was cloned into the *Bss*HII and *Pst*I site of pHAL1-D1.3scFab. For the construction of pHAL1-D1.3scFab+2, two codons were added and the PCR product was cloned into the BssHII and *Pst*I site as described. Both cysteines which connect both antibody chains were deleted to construct pHAL1D1.3 C. The cysteine of the LC was deleted by PCR primer directed mutagenesis and assembled with by a second PCR in which the cysteine of the Fd fragment was deleted. The PCR-product was cloned into the *Bss*HII and *Not*I site. Transformations of XL1-Blue MRF' (Stratagene, Amsterdam, Netherlands) with the constructs were done by electroporation according to the manufacturers' instruction. All steps in vector construction and antibody cloning were confirmed by sequencing of the affected regions.

### pOPE101 scFab production vectors

The D1.3scFv, D1.3Fab and D1.3scFabΔC DNA was were cloned from the vectors pHAL1-D1.3scFv, pHAL1-D1.3Fab and pHAL1-D1.3scFabΔC into the *E. coli *expression vector pOPE101-XP using the restriction sites *EcoR*I and *Not*I. pOPE101-XP is a modification of pOPE101-215yol [29] with the scFv coding sequence replaced by the short DNA sequence containing stop codons in all three reading frames. This vector contains a myc-tag and a his-tag for detection and purification.

### pPICZ-α scFab vectors for the expression in *P. pastoris*

D1.3scFabΔC DNA was cloned from pOPE101-D1.3Fab into the *P. pastoris *expression vector pPICZaA (Invitrogen, Karlsruhe, Germany) using the restriction sites *Xba*I and *Xho*I.

### Antibody phage production

50 mL 2xTY medium [29] + 100 μg/mL ampicillin + 100 mM glucose were inoculated with an overnight culture to O.D._600 _= 0.1. Bacteria were grown to O.D._600 _= 0.4 – 0.5 at 37°C and 250 rpm. 2 mL XL1-Blue MRF' (Stratagene) (~1 × 10^9 ^bacteria) were infected with 2 × 10^10 ^helper phage M13K07 (Stratagene) or Hyperphage [26,27,31], incubated at 37°C for 30 min without shaking, followed by 30 min at 250 rpm. The infected cells were harvested by centrifugation for 10 min at 3220 × g and the pellet was resuspended in 30 mL 2xTY + 100 μg/mL ampicillin + 50 μg/mL kanamycin. Phage were produced at 30°C and 250 rpm for 16 h. Cells were pelleted for 10 min at 10000 × g. Phage in the supernatant were precipitated with 1/5 volume of 20% PEG/2.5 M NaCl solution for 1 h on ice with gentle shaking before being pelleted 1 h at 10000 × g at 4°C. The precipitated phage were resuspended in 10 mL phage dilution buffer (10 mM TRIS, 20 mM NaCl, 2 mM EDTA, the pH was adjusted to pH 7.5 with HCl), precipitated again with 1/5 volume of PEG solution as given above for 20 min on ice and pelleted 30 min at 10000 × g at 4°C. The precipitated phage were resuspended in 300 μL phage dilution buffer and cell debris was pelleted by additional centrifugation for 5 min at 15400 × g at 20°C. The supernatant containing the antibody phage were stored at 4°C. Phage titration (cfu/mL) was done according to Koch *et al*. [40] with one modification: the infected bacteria were pipetted directly onto LB agar plates, omitting nitrocellulose sheets.

### Production of soluble antibody fragments using the phage display vector pHAL1

Soluble antibody fragments were produced in shaking flasks or microtiter plates (MTPs). For expression in shaking flasks, 100 mL 2xTY medium + 100 μg/mL ampicillin + 100 mM glucose were inoculated 1:20 with an overnight culture of XL1-Blue MRF' containing the appropriate vector and grown at 37°C and 250 rpm for 2 h. Bacteria were harvested by centrifugation for 20 min at 3900 × g. The pellet was resuspended in 100 mL 2xTY + 100 μg/ml Ampicillin + 20 μM IPTG at 30°C and 250 rpm overnight. 13 mL PBS (phosphate buffered saline [39]) containing 1% Tween20 were added and incubated at 30°C and 350 rpm for additional 3.5 h. Cells were separated from the antibody containing supernatant by centrifugation for 10 min at 7000 × g. For expression in MTPs 200 μl 2xTY medium + 100 μg/mL ampicillin + 100 mM glucose was inoculated with 10 μL overnight culture and grown at 37°C and 1400 rpm for 2 h. Bacteria were harvested by centrifugation for 10 min at 2500 × g. The pellet was resuspended in 200 μl 2xTY + 100 μg/mL Ampicillin + 20 μM IPTG at 30°C and 1400 rpm overnight. 50 μL PBS with 1% Tween20 were added and incubated at 30°C and 1400 rpm for additional 3.5 h. Cells were separated from the antibody containing supernatant by centrifugation for 10 min at 3200 × g.

### Production of soluble antibody fragments using the vector pOPE101-XP

Various D1.3 antibody formats were produced in shaking flasks according to Dübel *et al*. [41]. Briefly, 300 mL 2xTY + 100 μg/mL glucose + 100 μg/mL ampicillin were inoculated with an overnight culture yield to O.D._600 _< 0.1 and cultured at 30°C and 250 rpm. The induction was started by adjusting to 50 μM IPTG at O.D._600 _= 0.6 and shaken for 20 h. Bacteria were harvested by centrifugation for 10 min at 4400 × g at RT. Pellets were resuspended in 30 mL ice cold PE buffer, pH 8 (500 mM sucrose, 100 mM Tris, 1 mM EDTA) and incubated for 20 min on ice, interrupted by short vortexing every 5 min. Subsequently the bacteria were pelleted for 30 min at 30000 × g at 4°C. The supernatant (periplasmic fraction) was stored at -20°C. The pellet was resuspended in 30 mL ice-cold dH2O and incubated for 20 min on ice, interrupted by short vortexing every 5 min. Spheroblasts were centrifuged for 30 min at 30000 × g and 4°C. The supernatant (osmotic shock preparation) was stored at -20°C.

### Production of scFab in *P. pastoris*

Transformation of *P. pastoris (*KM71) (Invitrogen, Karlsruhe, Germany) was done according to the manufacturers recommendations. Positive clones were selected over YPD-Zeocin (100 μg/mL). To further select transformed clones for good antibody secretion a screening by 96 deep well (Greiner, Flacht, Germany) expression was performed using 500 μL BMGY medium, pH 6.0 [42] for 24 h at 30°C and 350 rpm. Protein synthesis was induced by changing the media to 500 μL BMMY, pH 6.0 containing 1% MeOH [42] and further incubation for 48 hours. Supernatant was harvested by centrifugation for 10 min, 1500 × g and analysed by antigen ELISA (using Protein L-HRP for detection). Identified clones showing high antibody secretion were expressed in 2 L shake flask containing 500 mL BMGY media which was inoculated to an OD_600 _0.5 and grown overnight. Protein production was induced by exchanging the media after 24 h with BMMY containing 1% MeOH and further incubation for 48 h, before collecting the supernatent by centrifugation for 10 min and 1500 × g.

### Protein L purification of soluble antibody fragments

Soluble antibody fractions from *E. coli *and *P. pastoris *expression were purified by affinity chromatography using a Protein L column (Pierce, Bonn, Germany). After loading, the column was washed with 10 column volumes PBS, pH 7.4. Antibody fragments were eluted with glycine buffer 0.1 M, 0.15 M NaCl, pH 2.6 with immediate neutralization by TRIS/HCl buffer 1 M, pH 9.0. Fractions were stored at -20°C.

### Immobilized metal affinity chromatography (IMAC) purification of soluble antibody fragments

Soluble antibody fragments were purified from *E. coli *derived material by affinity chromatography using IMAC. Chromatography using 1 mL Chelating Sepharose Fast Flow (Amersham Biosciences, Freiburg, Germany) was done according to the manufacturers' instruction using 10 mM imidazol containing buffer (20 mM Na2 HPO4, 0.5 M NaCl, 10 mM imidazol) when loading the beads with the protein solution, using 1× time 10 mM imidazol and 3× times 50 mM imidazol buffer for washing and PBS, pH 7.4 with 100 mM EDTA for the elution of the 6 × histidine tagged proteins.

### Antigen binding ELISA

Microtiter plates (Costar, Cambridge, USA) were coated with 100 ng hen egg white lysozyme in 100 μL 0.1 M NaHCO3, pH 9.6 per well over night at 4°C. Coated wells were washed 3× with PBS and blocked with 2% (w/v) skim milk powder in PBS for 1.5 h at RT, followed by 3× washing with PBS. Antibody phage or soluble antibody fragments were diluted in 100 μL blocking solution and incubated for 1.5 h, followed by 5× washing with PBST (PBS + 0.1% (v/v) Tween 20). Bound antibody phage were detected with mAb anti-M13 conjugated with HRP (Amersham Biosciences, Freiburg, Germany) (1:5000), soluble antibody fragments were detected either with mAb mouse anti-Strep-tag (Qiagen, Hilden, Germany) (1:10000) and mAb goat anti-mouse conjugated with HRP (1:50000), with mAb anti-myc tag (9E10 hybridom) (1:25) and mAb goat anti-mouse conjugated with HRP (1:50000) or with protein L conjugated with HRP (Pierce) (1:10000) and visualised with TMB (3,3',5,5'-tetramethylbenzidine) substrate. The staining reaction was stopped by adding 100 μL 1 N sulphuric acid. The absorbances at 450 nm and scattered light at 620 nm were measured and the 620 nm reference was subtracted using a SUNRISE microtiter plate reader (Tecan, Crailsheim, Germany).

### SDS-PAGE and Immunoblot

Antibody phage or soluble antibody fragments were separated by SDS-PAGE and blotted onto PVDF membrane. The membrane was blocked with 2% (w/v) skim milk powder in PBST for 1 h at RT. For the detection of antibody phage, the minor coat protein pIII was detected with 1:1000 diluted mouse mAb anti-pIII (Mobitec, Göttingen, Germany) for 1.5 h at RT, followed by 2× washing with PBST. Goat anti-mouse (Fc specific) (Sigma, Taufkirchen, Germany) conjugated with AP 1:1000 diluted was used for detection and visualised by NBT/BCIP. For the detection of soluble antibody fragments the mAb mouse anti-myc (supernatant 9E10 1:25) was used as primary antibody and goat anti-mouse conjugated with AP (Sigma, Taufkirchen, Germany) (1:2000) was used as secondary antibody.

### Gel filtration

Molecular sizes of purified scFabs were evaluated by size exclusion chromatography on a calibrated Superdex 200 10/300 GL column (Amersham Bioscience) with PBS, pH 7.4, at a flow rate of 1 mL/min. Samples were fractionated and stored for further ELISA analysis at 4°C.

### Affinity determination

Affinity measurements were done using surface plasmon resonance (SPR). The analysis was performed with a Biacore2000 (Biacore, Sweden) using a CM5 chip. Lysozyme and BSA were bound on flow cells via amine coupling. The flow rate was 30 μl/min at 25°C using HEPES buffer [39]. Sample concentration of D1.3 antibody variants loaded on flow cells ranged from 6 × 10^-8 ^to 5 × 10^-9 ^M. The curve fitting was performed and the kinetics were analyzed using the Biacore software package.

## Authors' contributions

MH drafted the manuscript and participated in the design and coordination of the study. TJ helped to draft the manuscript and participated in the design of both the molecular variants and the study. CM performed the *Pichia pastoris *experiments. BV performed the gelfiltration analysis. MB, AM, MIK and DM constructed the plasmids and made the *E. coli *phage display and production experiments. SD conceived the molecular design, organised funding and helped to draft the manuscript, designed and coordinated the study. All authors read and approved the final manuscript.
